# Supplementary material for: Redefining and estimating the early-phase reproduction ratio for epidemic outbreaks in spatially structured populations
Source: PLoS Comput Biol. 2026 Jul 1;22(7):e1014425. doi: 10.1371/journal.pcbi.1014425 (PMC13349308; doi:10.1371/journal.pcbi.1014425)
Supplement: S1 Appendix — This appendix contains the supplementary figures and methods. Fig A, Comparison of total and outbreak reproduction ratios. Fig B, Total error in risk estimation linked to urbanisation. Fig C, Total error stratified by country. Fig D, Numerical validation of epidemic probability estimates. Fig E, Effect of spatial aggregation on Rob estimates by comparing ADM-1 and ADM-2 spatial resolutions in Italy. Fig F, Numerical validation under overdispersed secondary infections. Fig G, Sensitivity of Rob to random noise and systematic bias in spatial contact data in Italy. Supplementary Methods include numerical validation of epidemic probability estimates, analytical derivation of Rob for overdispersed secondary infections, and analytical derivation of non-exponential infectious periods. (PDF) [file pcbi.1014425.s001.pdf]

S1 Appendix. Supplementary information for Redefining and estimating the early-phase reproduction ratio for epidemic outbreaks in spatially structured populations

Boxuan Wang[1] and Eugenio Valdano[1,*].

[1] *Sorbonne Université, INSERM, Institut Pierre Louis d'Epidémiologie et de Santé Publique, Paris, France.*

[*] *Corresponding author* eugenio.valdano@inserm.fr

Supplementary Figures

A Comparison of total and outbreak reproduction numbers 2
B Total error in risk estimation linked to urbanisation 2
C Total error stratified by country 3
D Numerical validation of epidemic probability estimates 3
E Effect of spatial aggregation on R^{ob} estimates by comparing different spatial aggregations 4
F Numerical validation of epidemic probability estimates in the case of overdispersed secondary infections 5
G Sensitivity of R^{ob} to random noise and systematic bias 6

Supplementary Methods

S1 Numerical validation of epidemic probability estimates **2**

S2 Analytical derivation of R^{ob} for overdispersed secondary infections **7**

S3 Analytical derivation for non-exponential infectious periods **7**

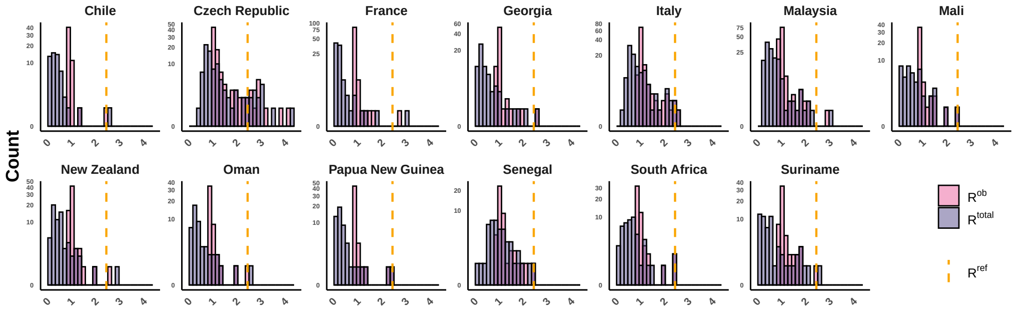

Figure A: **Comparison of total and outbreak reproduction numbers.** Histograms comparing the distributions of the total reproduction number (R^{tot}, grey bars) and the outbreak reproduction ratio (R^{ob}, purple bars) for a scenario where $R^{\text{ref}} = 2.5$. The dashed orange line indicates the global reference.

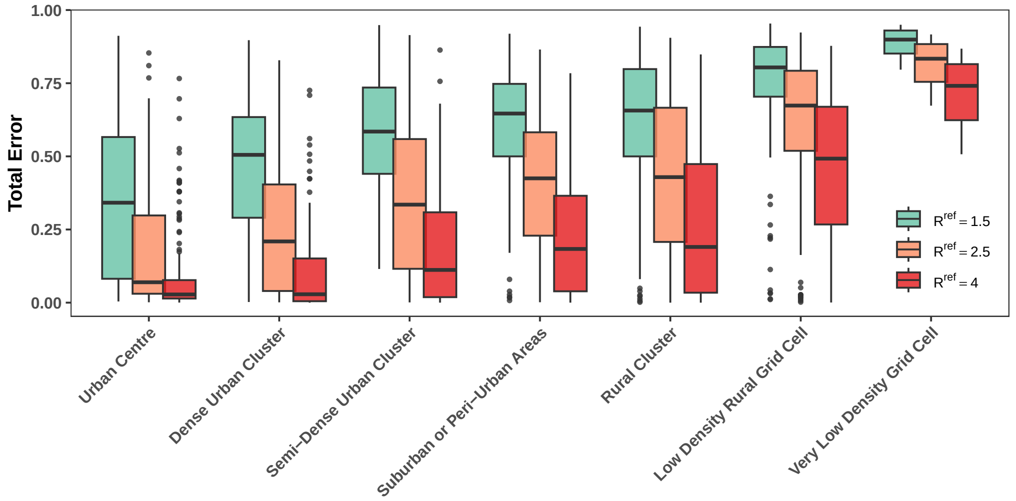

Figure B: **Total error in risk estimation linked to urbanisation.** Relative total error (defined as the normalized difference between R^{ob} and R^{tot}) stratified by urbanization level for three transmission intensities (R^{ref}). Similar to the local error, the total error tends to increase as urbanisation level decreases (from urban to rural), indicating that Rtot also underestimates risk in rural areas.

S1 Numerical validation of epidemic probability estimates

To validate the epidemic probability estimates derived from the multitype branching process framework, we compared theoretical predictions with numerical simulations based on spatial contact data for Italy.

Epidemic probabilities were estimated as the fraction of sampled stochastic realizations ($2,000$ in total) in which the outbreak size exceeded a given threshold, exploring different threshold values and seeding locations. Results in Fig D demonstrate close agreement between theory and simulations across a wide range of outbreak size cutoffs, with convergence observed even for relatively small outbreak sizes.

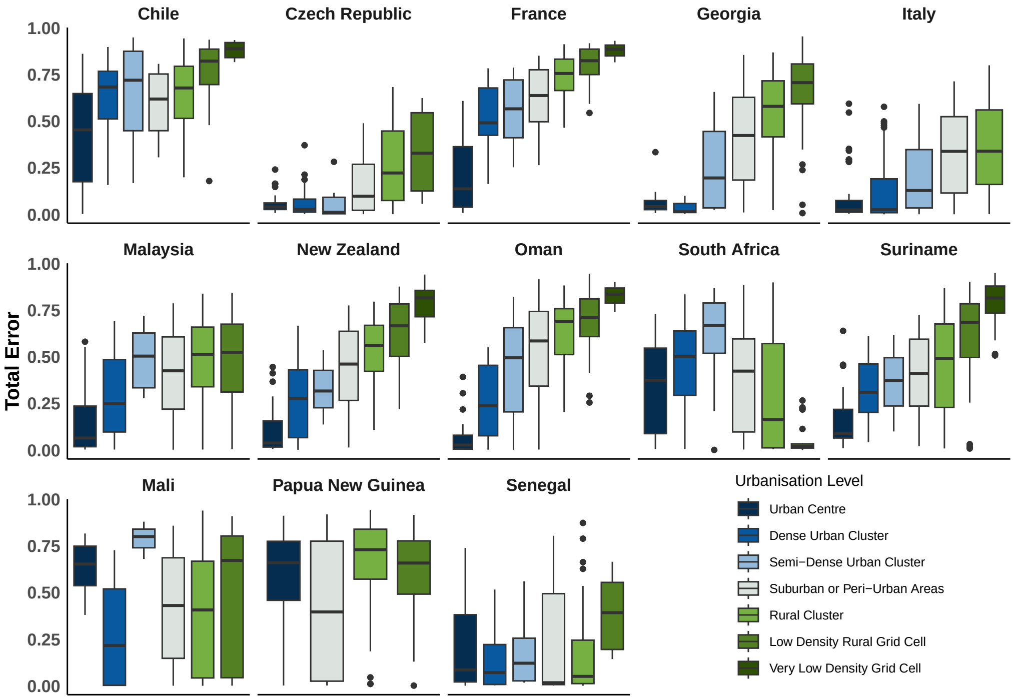

Figure C: **Total error stratified by country.** Total error stratified by country income group for the scenario $R^{\text{ref}} = 2.5$. Top: In upper-middle and high-income economies, rurality remains a predictor of estimation bias even when using R^{tot}. Bottom: In low and lower-middle-income economies, the relationship shows higher variability.

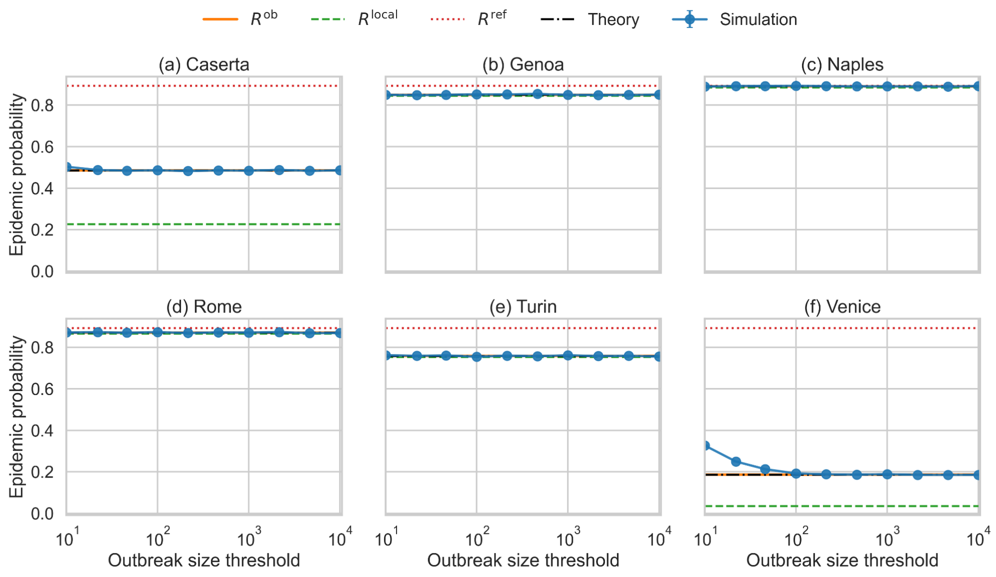

Figure D: **Numerical validation of epidemic probability estimates.** The figure compares epidemic probabilities estimated from 2,000 stochastic simulations with $R^{\text{ref}} = 2.5$. Dots represent estimated probabilities, bars indicate 95% confidence intervals. The horizontal red line indicates the theoretically-predicted values of the epidemic probability. The x-axis represents the cut-off size used to discriminate epidemics from minor outbreaks. Synthetic epidemic data were generated for Italy, using Colocation Maps (see Methods). Different plots test different locations for the initial pathogen introduction.

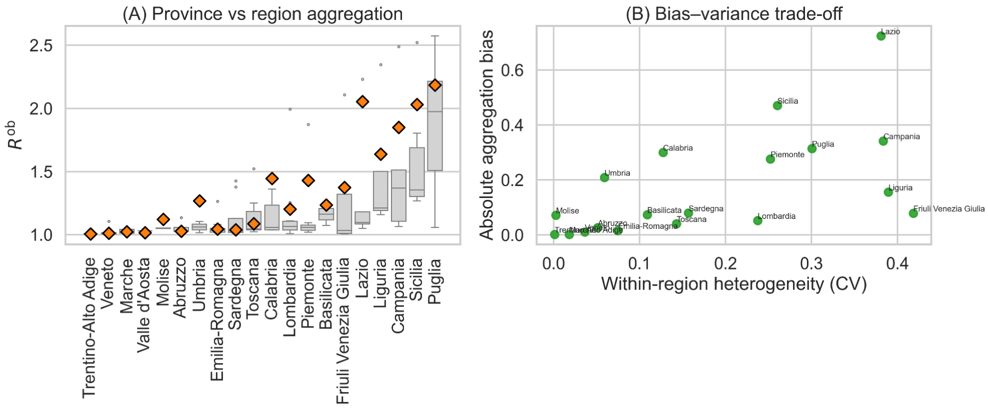

Figure E: **Effect of spatial aggregation on R^{ob} estimates by comparing regional (ADM 1) and province-level (ADM 2) values for Italy. a.** Distribution of province-level R^{ob} values within each region (gray dots), compared with the corresponding values obtained after aggregating the transmission matrix at the level of regions (diamonds). Substantial within-region heterogeneity is observed, and the aggregated region-level estimates can deviate from the central tendency of the province-level distributions, indicating that spatial aggregation does not simply average local outbreak risks but can alter their effective representation.**b.** Absolute aggregation bias, defined as the absolute difference between the region-level R^{ob} and the mean province-level R^{ob} within the same region, plotted against the within-region coefficient of variation (CV) of province-level R^{ob}. Regions with higher within-region heterogeneity may exhibit larger aggregation bias, illustrating the impact of spatial aggregation on the representation of epidemic risk. The data used are the same as in Fig D. These results illustrate that coarser spatial resolutions not only smooth out spatial variations in epidemic risk but may also bias the accuracy of coarse-level risk averages when risk heterogeneity is substantial. Therefore, the appropriate spatial scale should be chosen based on the degree of heterogeneity and the level at which epidemiological decisions are made.

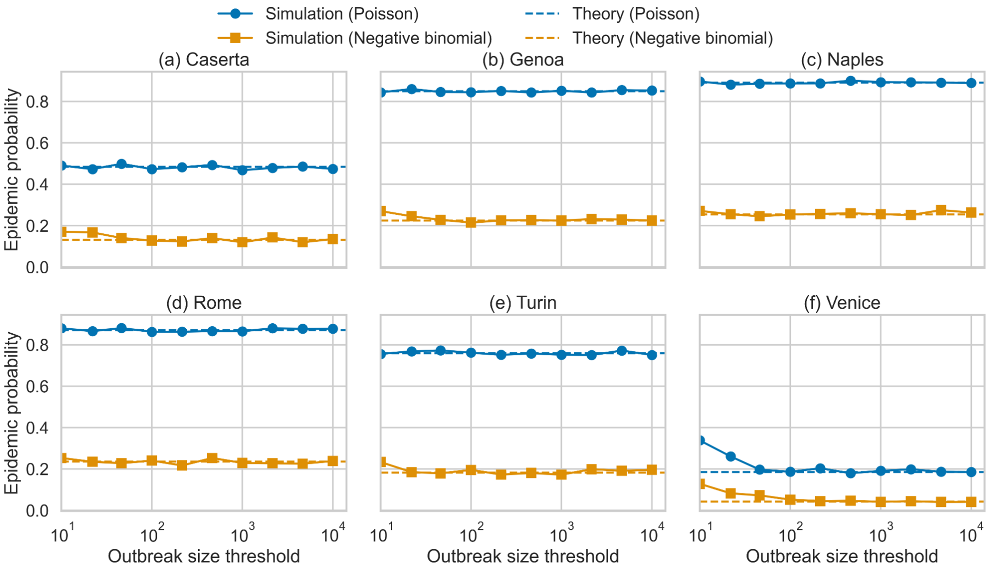

Figure F: **Numerical validation of epidemic probability estimates in the case of overdispersed secondary infections.** The figure compares epidemic probabilities estimated from 2,000 stochastic simulations under both Poisson- and negative-binomially-distributed secondary infections, with $R^{\text{ref}} = 2.5$ and $\omega = 5$ (the latter parameter being the overdispersion parameter of negative-binomial distribution, see Eq. 6 of the main text). Dots represent estimated probabilities, while bars indicate 95% confidence intervals. Horizontal lines indicate the theoretically-predicted values of the epidemic probability for the Poisson (black) and negative-binomial (blue) cases, respectively. The x-axis represents the cut-off size used to discriminate epidemics from extinct minor outbreaks. Synthetic epidemic data were generated for Italy using Colocation Maps (see Methods of the main text). Different plots test different locations for the initial pathogen introduction. The same level of agreement is observed in the presence of overdispersion, indicating that the framework remains valid beyond the Poisson assumption.

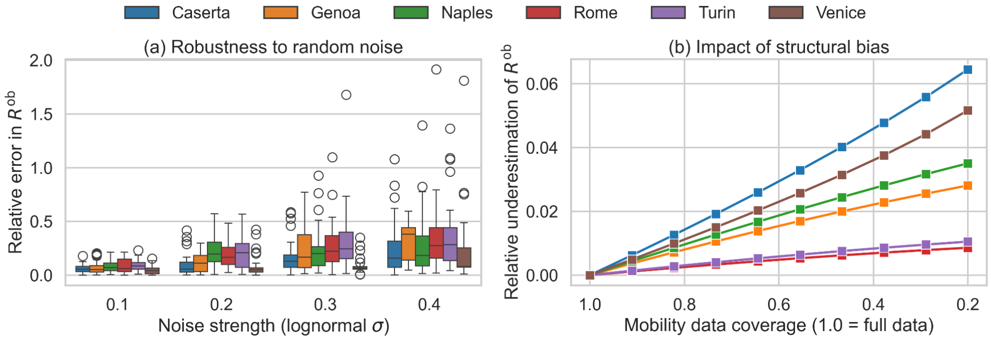

Figure G: **Sensitivity of $R^{\rm ob}$ to random noise and systematic bias in spatial contact data in Italy. a.** Random noise was introduced by multiplying each element of the colocation matrix by independent lognormal random variables with parameters $\mu = 0, \sigma$. For each noise level, 30 independent realizations were generated, and the distribution of relative errors in $R^{\rm ob}$ is shown for selected regions. Synthetic contact data were generated for Italy using Colocation Maps (see Methods). **b.** Systematic bias was modeled by uniformly scaling all off-diagonal elements of the reproduction operator by a factor between 0.2 and 1.0, representing underestimation of inter-regional mobility. The resulting relative underestimation of $R^{\rm ob}$ is shown as a function of data coverage. These results show that $R^{\rm ob}$ is robust to random noise in contact data, while systematic biases lead to predictable and interpretable deviations.

S2 Analytical derivation of $R^{\rm ob}$ for overdispersed secondary infections

We start by noting that Eq. (7) through Eq. (14), as well as the identity in Eq. (18) of the main text hold in general. We then assume that secondary infections are negative-binomially distributed with overdispersion ω, so that $\mathbb{E}[X_{i\to j}] = R_{ji}$ and $\mathrm{Var}[X_{i\to j}] = R_{ji}\,(1+\omega R_{ji})$, leading to a probability generating function $g(z|R_{ji},\omega) = [1+\omega R_{ji}(1-z)]^{-1/\omega}$ and Eq. (5) of the main text for epidemic probabilities.

We then compute the logarithmic derivative of the probability generating function:

$$\psi(z|r,\omega) = \frac{g'(z|r,\omega)}{g(z|r,\omega)} = \frac{r}{1+\omega r(1-z)}, \tag{S1}$$

from which one can see that the Poisson case ($\omega = 0$) correctly gives $g'(z|r) = rg(z|r)$, as used in the main text.

Using this in Eq. (14) of the main text, we can write

$$E_{ij}^{+} = \frac{1}{p_i}\left\{R_{ji} - (1-p_j)\psi(1-p_j|R_{ji},\omega)\prod_k g(1-p_k|R_{ki},\omega)\right\} \tag{S2}$$

$$= \frac{1}{p_i}\left[R_{ji} - \frac{(1-p_i)(1-p_j)R_{ji}}{1+\omega R_{ji}p_j}\right]. \tag{S3}$$

To get to the last line we used Eq. (5) of the main text and the specific form of the probability generating function of the negative-binomial distribution.

Now that we have E_{ij}^{+} we can use Eq. (18) of the main text to get E_{ij}^{-} and then compute the expression of $R^{\rm ob}$, which is

$$R_i^{\rm ob} = \sum_j \left(E_{ij}^{+} - E_{ij}^{-}\right) = \frac{1}{p_i}\sum_j R_{ji}p_j\frac{1+\omega R_{ji}}{1+\omega R_{ji}p_j}. \tag{S4}$$

The correction to the Poisson case (Eq. (3) of the main text) can be made explicit:

$$R_i^{\rm ob} = R_{\mathrm{Poisson},i}^{\rm ob} + \frac{\omega}{p_i}\sum_j \frac{R_{ji}^2 p_j(1-p_j)}{1+\omega R_{ji}p_j}. \tag{S5}$$

First, the correction correctly disappears in the Poisson limit ($\omega \to 0$). Second, the correction term is nonnegative, so, at least at first order in ω (small overdispersion), overdispersion increases $R^{\rm ob}$ with respect to the Poisson case, which is compatible with the known impact of heterogeneity on epidemic risk.

S3 Analytical derivation for non-exponential infectious periods

The infectious period, and in particular its distribution, is known to affect epidemic dynamics across all phases. Here, we focus on the early epidemic phase and use the branching-process formalism. Within this framework, an arbitrary infectious-period distribution can be incorporated by modifying the distribution of secondary infections as follows. Let $\tau \in [0,\infty)$ be the stochastic variable representing the infectious period, with probability density function h. Let $\beta \geq 0$ be the rate at which secondary infections are generated during the infectious period. For simplicity, we first consider a single population with reproduction ratio r; the same construction can then be applied to any transmission route $i \to j$ in the multitype branching process. For consistency, one

must have $\beta = r/\bar{\tau}$, where $\bar{\tau}$ is the mean infectious period. Conditioned on τ, the number of secondary infections X is Poisson-distributed, with mean $\beta\tau$. So one can recover the distribution of secondary infections f by marginalizing over all the possible values of τ:

$$f(X) = \int_0^\infty \mathrm{d}\tau \, h(\tau) \frac{e^{-\beta\tau} (\beta\tau)^X}{X!}. \tag{S6}$$

Now, let us assume that the infectious period is modeled as a Gamma distribution, with mean $\bar{\tau}$ and shape parameter k, $k = 1$ being the exponential distribution. For $k > 1$ the distribution has a peak for $\tau > 0$, and that is why it is a common and versatile model for realistic infectious period distributions. Inserting the Gamma distribution in Eq. (S6) one can compute the integrals and gets

$$f(X) = \binom{X + k - 1}{X} \left(\frac{r}{r+k}\right)^X \left(\frac{k}{r+k}\right)^k, \tag{S7}$$

and this is a negative binomial distribution with mean r and overdispersion $\omega = 1/k$. This proves that, in our formalism, a generic (Gamma-distributed) infectious period distribution is equivalent to a negative-binomially-distributed number of secondary infections, with their overdispersion being tuned by the shape of the underlying Gamma distribution. The Poisson distribution ($\omega = 0$) is recovered when $k \to \infty$, which means when the infectious period can be assumed to be constant, or almost constant. Conversely, heterogeneous infectious period lengths map to overdispersed distributions for secondary infections.
