# Supplementary material for: Redefining and estimating the early-phase reproduction ratio for epidemic outbreaks in spatially structured populations
Source: PLoS Comput Biol. 2026 Jul 1;22(7):e1014425. doi: 10.1371/journal.pcbi.1014425 (PMC13349308; doi:10.1371/journal.pcbi.1014425)
Supplement: Attachment — Submitted filename: response_to_reviewers.pdf [file pcbi.1014425.s004.pdf]

EDITOR AND JOURNAL COMMENTS

Journal Requirements

1) Please ensure that the CRediT author contributions listed for every co-author are completed accurately and in full.
At this stage, the following Authors/Authors require contributions: Boxuan Wang, and Eugenio Valdano. Please ensure that the full contributions of each author are acknowledged in the "Add/Edit/Remove Authors" section of our submission form.
The list of CRediT author contributions may be found here:
https://journals.plos.org/ploscompbiol/s/authorship#loc-author-contributions

Done.

2) We ask that a manuscript source file is provided at Revision. Please upload your manuscript file as a .doc, .docx, .rtf or .tex. If you are providing a .tex file, please upload it under the item type u2018LaTeX Source Fileu2019 and leave your .pdf version as the item type u2018Manuscriptu2019.

Done.

3) Please provide an Author Summary. This should appear in your manuscript between the Abstract (if applicable) and the Introduction, and should be 150-200 words long. The aim should be to make your findings accessible to a wide audience that includes both scientists and non-scientists. Sample summaries can be found on our website under Submission Guidelines:
https://journals.plos.org/ploscompbiol/s/submission-guidelines#loc-parts-of-a-submission

Done.

4) Please upload all main figures as separate Figure files in .tif or .eps format. For more information about how to convert and format your figure files please see our guidelines:
https://journals.plos.org/ploscompbiol/s/figures

Done.

5) We have noticed that you have uploaded Supporting Information files, but you have not included a list of legends. Please add a full list of legends for your Supporting Information files after the references list.

Done.

6) Some material included in your submission may be copyrighted. According to PLOSu2019s copyright policy, authors who use figures or other material (e.g., graphics, clipart, maps) from another author or

copyright holder must demonstrate or obtain permission to publish this material under the Creative Commons Attribution 4.0 International (CC BY 4.0) License used by PLOS journals. Please closely review the details of PLOSu2019s copyright requirements here: PLOS Licenses and Copyright. If you need to request permissions from a copyright holder, you may use PLOS's Copyright Content Permission form. Please respond directly to this email and provide any known details concerning your material's license terms and permissions required for reuse, even if you have not yet obtained copyright permissions or are unsure of your material's copyright compatibility. Once you have responded and addressed all other outstanding technical requirements, you may resubmit your manuscript within Editorial Manager.
Potential Copyright Issues:

- Figure 1C and 4A: Please (a) provide a direct link to the base layer of the map (i.e., the country or region border shape) and ensure this is also included in the figure legend; and (b) provide a link to the terms of use / license information for the base layer image or shapefile. We cannot publish proprietary or copyrighted maps (e.g. Google Maps, Mapquest) and the terms of use for your map base layer must be compatible with our CC BY 4.0 license.
Note: if you created the map in a software program like R or ArcGIS, please locate and indicate the source of the basemap shapefile onto which data has been plotted.
If your map was obtained from a copyrighted source please amend the figure so that the base map used is from an openly available source. Alternatively, please provide explicit written permission from the copyright holder granting you the right to publish the material under our CC BY 4.0 license.
If you are unsure whether you can use a map or not, please do reach out and we will be able to help you.
The following websites are good examples of where you can source open access or public domain maps:
* U.S. Geological Survey (USGS) - All maps are in the public domain. (http://www.usgs.gov)
* PlaniGlobe - All maps are published under a Creative Commons license so please cite u201cPlaniGlobe, http://www.planiglobe.com, CC BY 2.0u201d in the image credit after the caption. (http://www.planiglobe.com/?lang=enl)
* Natural Earth - All maps are public domain. (http://www.naturalearthdata.com/about/terms-of-use/).

Done. We used Natural Earth maps.

7) Please amend your detailed Financial Disclosure statement. This is published with the article. It must therefore be completed in full sentences and contain the exact wording you wish to be published.
State what role the funders took in the study. If the funders had no role in your study, please state: "The funders had no role in study design, data collection and analysis, decision to publish, or preparation of the manuscript.".
If you did not receive any funding for this study, please simply state: u201cThe authors received no specific funding for this work.u201d

Done.

REVIEWER 1

In the study by Wang and Valdno, the authors define an outbreak reproduction number that takes into account spatial heterogeneity. They use colocation data and show that various locations have spatial heterogeneity I their transmission potential. Furthermore, they apply their reproduction number to early outbreak data to estimate locations at higher risk. The study is interesting, rigorous and well-written overall. I do have some questions and comments that should be answered before it is worthy of publication, in my opinion.

- **The metrics used to investigate the usefulness of the proposed method. As you state, the main point of the paper is "…quantifying epidemic outbreak risk in spatially structured populations…". However, there is no direct comparison between your and other methods, or even just a direct quantification of the precision of your method, for the probability of outbreak estimate. I think that it is worth performing simulations and using real data to see how well you can estimate outbreak probabilities under various conditions, rather than just estimating reproduction numbers.**

To address this point, we validated the estimates of the probability of outbreak (epidemic probability) from the multitype branching process theory against numerical simulations. We used Italy as validation, using colocation maps for that country. We compared theoretical predictions with the estimated epidemic probability computed as the fraction of stochastic realizations (2,000 realizations in total) in which the outbreak size exceeded a given threshold, and explored different values of the threshold.We also explored different locations for the pathogen introduction. The results of this analysis are presented in Supplementary Figure 4, reported here below. The theoretically predicted epidemic probabilities match the simulation-based estimates across a wide range of seed locations and outbreak size thresholds, notably converging even for small outbreaks (fewer than fifty cases. Full details of the simulation procedure are provided in the Supplementary Information (Supplementary Method Section S1).

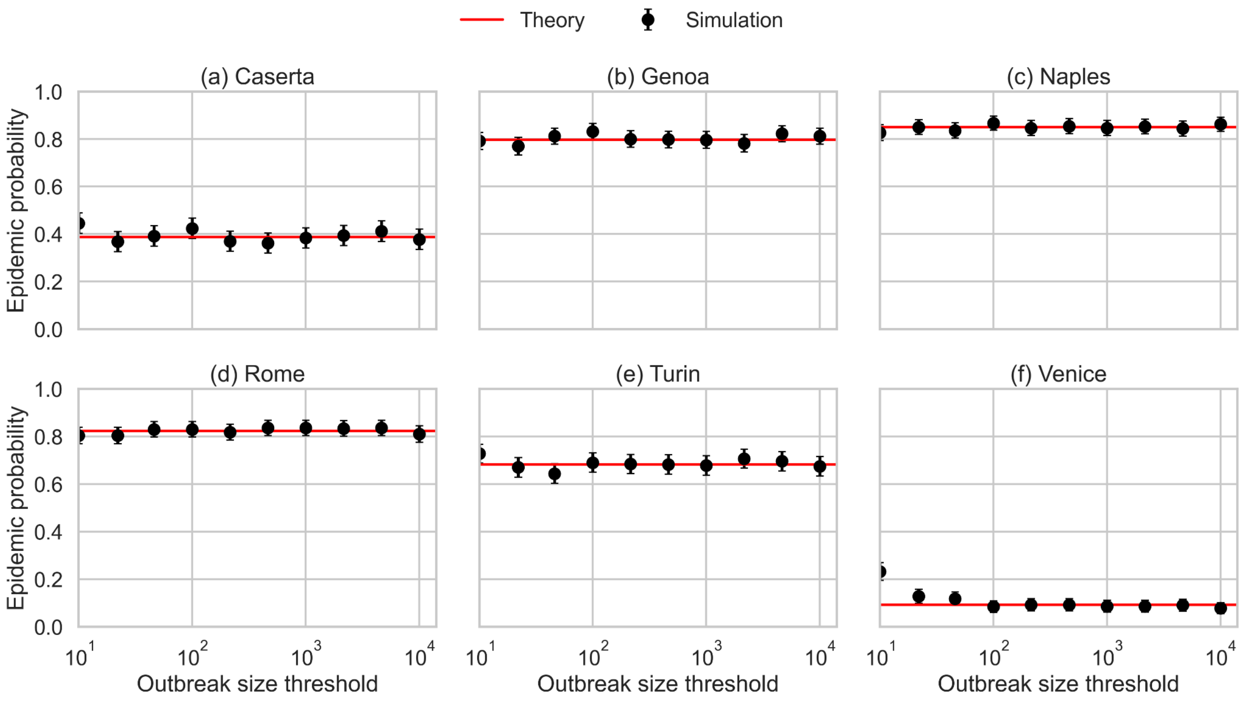

Supplementary Fig. S4. Numerical validation of epidemic probability estimates. *The figure compares epidemic probabilities estimated from 2,000 stochastic simulations.Dots represent estimated probabilities, bars indicate 95% confidence intervals. The horizontal red line indicates the theoretically-predicted values of the epidemic probability. The x-axis represents the cut-off size used to discriminate epidemics from minor outbreaks. Synthetic epidemic data were generated for Italy, Colocation Maps (see Methods, "Spatial contact data to model the reproduction operator"). Different plots test different locations for the initial pathogen introduction.*

- **If you have several levels of spatial resolution (e.g. neighborhood, city, county), how would you choose the right one? Is there some criterion in your framework to quantify this bias-variance trade-off? This should probably be demonstrated, but at least discussed.**

We agree that the choice of spatial resolution is important when estimating epidemic risk in practical public-health settings. Our framework is formally agnostic to the chosen resolution, but the reliability and operational relevance of the resulting estimates inevitably depend on the granularity and quality of the available data. A systematic investigation of this issue lies beyond the scope of the present study and would require disease- and context-specific analyses aimed at identifying an optimal spatial scale in a data-driven manner. Here, however, we have added a first analysis of this question using Italy as a case study, comparing two levels of spatial

aggregation: provinces (ADM-2, used throughout the study) and regions (ADM-1). The results are presented in Supplementary Fig 5 (also reported here below), which compares estimates of R^{ob} at the level of provinces with the values of R^{ob} estimated of the level of the region they belong to. Panel (A) shows that there is substantial within-region heterogeneity in province-level R^{ob} values, which is smoothed out when the system is aggregated to the region level. Panel (B) quantifies the effect of aggregation by relating the absolute aggregation bias (defined as the absolute difference between the region-level R^{ob} and the mean province-level R^{ob}) to the within-region coefficient of variation of province-level R^{ob} in that region. Regions with higher internal heterogeneity may also exhibit larger aggregation bias. These results illustrate that coarser spatial resolutions not only smooth out spatial variations in epidemic risk but may also bias the accuracy of aggregated risk estimates, when risk heterogeneity is substantial. Therefore, the appropriate spatial scale should be chosen based on the degree of heterogeneity and the level at which epidemiological decisions are made. We have also added a dedicated discussion on this bias-variance trade-off and the criteria for selecting an appropriate spatial resolution in practical settings.

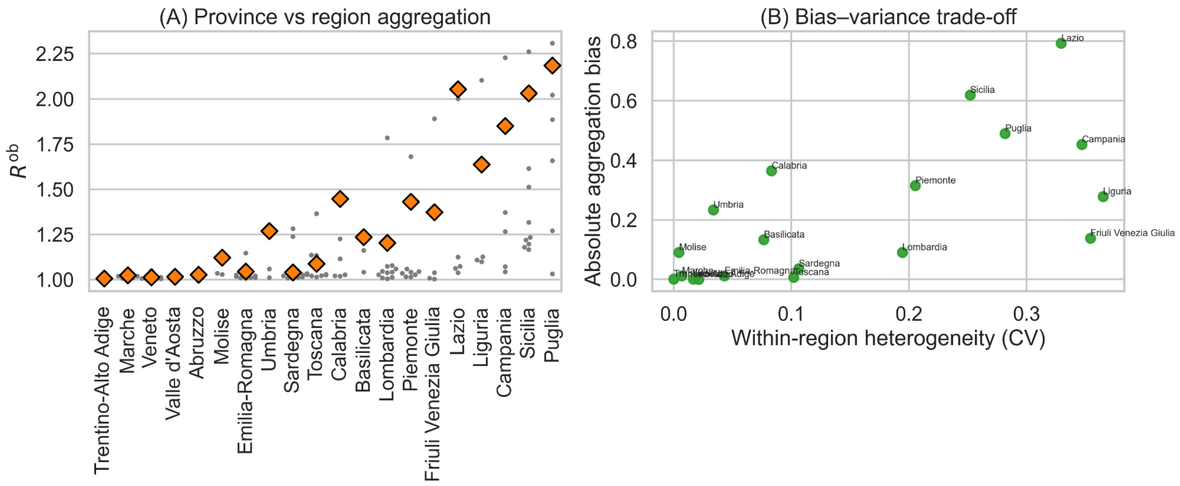

Supplementary Fig 5. Effect of spatial aggregation on Rob estimates by comparing regional (ADM 1) and province-level (ADM 2) values for Italy. *(A) Distribution of province-level Rob values within each region (gray dots), compared with the corresponding values obtained after aggregating the transmission matrix at the level of regions (diamonds). Substantial within-region heterogeneity is observed, and the aggregated region-level estimates can deviate from the central tendency of the province-level distributions, indicating that spatial aggregation does not simply average local outbreak risks but can alter their effective representation.(B) Absolute aggregation bias, defined as the absolute difference between the region-level Rob and the mean province-level Rob within the same region, plotted against the within-region coefficient of variation (CV) of*

province-level Rob. Regions with higher within-region heterogeneity may exhibit larger aggregation bias, illustrating the impact of spatial aggregation on the representation of epidemic risk. The data used are the same as in Supplementary Figure 4.

- **You explain why a Poisson process is reasonable, despite common overdispersion, because overdispersion may be dealt with by your stratification of the population. However, this does not include cases of superspreaders, even in mostly homogenous populations (e.g. a fast-food worker sneezing on hundreds of burgers every day). Although you somewhat discuss this, it is a bit downplayed in my opinion, since there is a lot of evidence that superspreaders may play a major role in outbreak dynamics. I think that some simulations showing that your framework holds when introducing superspreaders would be beneficial.**

Thank you for giving us the opportunity to illustrate the fact that our methodology is not limited to assuming a Poisson offspring distribution, while that is a simple yet effective choice (as we discuss in the manuscript). The derivation of can be R^{ob} carried out with a generic offspring distribution, and most interestingly, as the reviewer suggests, by assuming a negative binomial distribution, of which the Poisson distribution is a limiting case. We have updated the Methods and the Supplementary Information to include the calculations in the case of a negative binomial distribution, so that the Reader who is interested in estimating R^{ob} in the presence of superspreading events beyond those related to population stratification can do so. In addition, Supplementary Figure 6 now also validates theoretical estimates of the epidemic probability in the case of negative binomial distributions, proving that our methodology is correct even in the presence of overdispersion. The detailed derivations of R^{ob} from epidemic probabilities with a negative binomial distribution are provided in the Supplementary Method Section S2.

- **Are your estimates correct if the infectious period is not exponential? There are some works showing that the probability of an outbreak differs, and may be underestimated, if the infectious period is e.g. gamma distributed instead of exponential (which is quite common in real-world settings).**

We have expanded our theoretical derivation to address this point. While the branching process formalism does not explicitly feature an infectious period, it is possible to link the probability distribution of the infectious period to the probability distribution of the number of secondary cases. Notably, it is possible to model a gamma-distributed infectious period, which the reviewer

is right to point out as a realistic and widely used model, using negative-binomial-distributed secondary infections, which we now cover in our study (see previous point). We prove the link between infectious period and secondary infections in Supplementary Method Section 3, demonstrating that our framework is robust to deviations from exponential infectious-period assumptions, provided these effects are captured by means of an overdispersed offspring distribution.

- **What happens when the spatial contact data is noisy or biased, as may well be the case quite often? Perhaps worth pursuing in simulations, when the ground truth is known.**

To address this, we performed additional analyses to quantify the sensitivity of R^{ob} to both random noise and systematic bias in the spatial contact data, now reported in Supplementary Fig 7, again using Italy as a test case. For random noise, we applied multiplicative lognormal perturbations independently to each element of the reproduction operator. We found that R^{ob} is robust to such stochastic noise, with only small relative errors even at high noise levels. In contrast, systematic errors in mobility data leads to biased risk assessment. To prove this, we uniformly scaled all inter-community transmission terms (off-diagonal elements in the reproduction operator), representing incomplete observation spatial contacts. This results in a consistent underestimation of R^{ob}, which increases as data coverage decreases. Importantly, this bias is not uniform across regions: areas with lower local transmission potential (Rii) are more sensitive to missing inter-regional interactions, leading to larger errors. Overall, these results demonstrate that, expectedly, R^{ob} is robust to random noise, while systematic bias in estimating spatial contacts can substantially affect epidemic risk.

- **Are there other estimates of R that summarize local information from heterogenous groups? prior work is worth referring to and comparing against.**

We thank the reviewer for this suggestion. There is indeed prior work extending reproduction-number estimation to heterogeneous and spatial settings, notably through multi-type or spatial renewal-equation frameworks (e.g., Green et al., 2022; Trevisin et al., 2023). These approaches incorporate mobility or group structure and infer time-varying reproduction numbers from observed incidence data.

Our objective is different. Rather than estimating Rt from outbreak data, our framework focuses on the early stochastic phase of epidemic emergence, where incidence may be sparse or unavailable. In this regime, the key quantity is the probability of a major outbreak. Our definition

of R^{ob} connects heterogeneous structure to this outbreak probability, providing a complementary perspective to existing inference-based approaches.

To clarify this distinction, we have now revised the Introduction to explicitly position our framework with respect to existing spatial and heterogeneous Rt estimation methods. In particular, we now emphasize that existing approaches primarily focus on inferring transmission dynamics from incidence data once epidemic circulation has already been established, whereas our framework instead quantifies the probability that an early stochastic introduction event triggers a large-scale epidemic in a spatially structured population.

- **"We now show that Rob behaves consistently as a reproduction ratio by demonstrating that it reduces to known quantities in limiting cases, and has the same critical behavior. First, the epidemic threshold of the system (Rref = 1) correctly maps to Rob = 1." Does this have to be the case? Why limit yourself to this?**

This is not a restrictive assumption, but a necessary consistency requirement for any meaningful reproduction metric. In the single-population limit, the classical reproduction number R fully characterizes epidemic growth and outbreak probability. Therefore, any generalized quantity such as R^{ob} must reduce to R in this limit. If it did not, Rob would not have a clear epidemiological interpretation: it would disagree with a well-established quantity in the simplest possible setting, raising the question of what it actually represents. More broadly, requiring that R^{ob}=1 corresponds to the epidemic threshold ensures that it preserves the critical behavior of the system. This guarantees that R^{ob} is not introducing a new or incompatible notion of transmission, but rather provides a consistent extension of classical reproduction numbers to heterogeneous and stochastic settings. In this sense, the reduction of R^{ob} to known quantities in limiting cases is not a constraint, but a validation step: it ensures that the proposed quantity remains interpretable, comparable to existing metrics, and grounded in established epidemiological theory.

- **Why do you have consistent overestimation of R (as in figure 3)?**

Figure 3 shows that R^{ob} is generally overestimated from early surveillance data. This is not a flaw in the estimator, but from an intrinsic selection effect and survivor bias, which is known to affect early estimates of transmission and reproduction ratio. Specifically, in the early phase, many transmission chains undergo stochastic extinction even in supercritical regimes. Consequently, the realizations that survive to be observed are statistically more likely to be

those that experienced above-average early growth. In reality this is realized, for instance, through core groups (see, for example, 10.2807/ese.14.22.19227-en), i.e., subcommunities where transmission rates are higher than the general population and fuel initial persistence. This translates into a systematic upward bias in the inferred transmission intensity, as surviving outbreaks represent the upper tail of the growth distribution rather than the mean behavior. As the outbreak size increases, this selection effect disappears: the cumulative data become more representative of the underlying process, and the maximum-likelihood estimates converge toward their theoretical values. Figure 3 shows that this happens rather quickly as the outbreak size increases.

We now mention this aspect in the manuscript when describing Figure 3, as follows:

Expectedly, estimates were noisy and tended to overestimate R^{ob} for small outbreaks, reflecting a well-known selection effect whereby only transmission chains with above-average early growth avoid stochastic extinction and are therefore observed, a mechanism often associated with early transmission concentrated in higher-risk groups [44].

- **The order of the legend of figure 6b is not the same as figure 6a.**

We thank the reviewer for pointing this out. We have revised Figure 6b so that the legend order is consistent with Figure 6a, improving clarity and visual coherence across panels.

- **Figures are not consistently referred to (e.g. Figure 5 and Fig. 5), and I can’t see figure 6 mentioned in the text.**

We thank the reviewer for pointing out these oversights. We have addressed these issues as follows:

1. Consistency in Figure Citations: We have standardized the nomenclature for figure citations throughout the manuscript. Following standard editorial conventions, we now use "Figure" at the beginning of a sentence and "Fig." for all in-text and parenthetical references.
2. Citation of Figure 6: We have now explicitly integrated references to Figure 6 within the Results section under the subsection *"Application to early SARS-CoV-2 transmission in Canada"*.

REVIEWER 2

The paper is well written. The authors develop a metric that uses branching process theory and the Lambert W function to define a value that can be considered "What is the equivalent Reproduction number for a seed arising in this community (could be extended to any subtype) that takes into account connectivity with other places (types) that would have the same outbreak probability as if the community were isolated?"

I would encourage the authors to add a brief clarification distinguishing the Lambert-W solution from standard next-generation-matrix quantities such as the dominant eigenvalue (spectral radius) and eigenvector-based sensitivity measures. As written, a reader familiar with next-generation-matrix methods may wonder whether is intended to be a community-specific analogue of the spectral radius, or perhaps something close to a sensitivity measure derived from the associated left and right eigenvectors. My understanding is that these are different objects. The Lambert- expression arises in the scalar, isolated-community case, where the outbreak probability solves =1− ^− . In the spatially coupled system, by contrast, the relevant quantity is the multitype outbreak probability, obtained from the coupled fixed-point system, and _ ^ is then defined by mapping that seed-specific probability back through the scalar survival relation. In other words, _ ^ is a nonlinear, introduction(seed)-specific effective reproduction number, not a local dominant eigenvalue or a sensitivity derivative of the spectral radius of the next-generation matrix. This distinction seems especially important in the toy two-community example, where coupling can change _ and hence _ ^ even when the system-level spectral radius is unchanged. At the same time, there is a useful formal connection to standard next-generation-matrix theory in the homogeneous and below-threshold limits, where the manuscript shows that _ ^ collapses to _ and the dominant-eigenvector structure reappears. Making this distinction explicit would help readers connect the present framework to the standard NGM literature without conflating outbreak probability, invasion threshold, and eigenvector-based sensitivity analysis.

We thank the reviewer for this insightful comment. We agree that it is important to clearly distinguish R^{ob} from standard next-generation-matrix quantities such as the spectral radius and eigenvector-based sensitivity measures.

In our framework, R^{ob} is not a local analogue of the dominant eigenvalue, nor a sensitivity measure derived from eigenvectors. Instead, it is defined through a two-step construction: first, the outbreak probability for a given seed is obtained from the multitype branching process via the coupled fixed-point system; second, this seed-specific probability is mapped back to an equivalent scalar reproduction number using the survival relation of the single-type process (involving the Lambert W function). As a result, R^{ob} is a nonlinear, seed-specific effective reproduction number that encodes stochastic invasion dynamics, rather than expected growth rates.

By contrast, the spectral radius of the next-generation matrix characterizes the asymptotic growth rate and invasion threshold of the system as a whole, while eigenvectors describe sensitivity or contribution of

different groups to this growth. These are fundamentally different objects: R^{ob} is tied to outbreak probability, whereas the spectral radius is tied to expected growth.

We have now clarified this distinction in the manuscript. We also emphasize the connection between the two frameworks in limiting cases: in the homogeneous or near-threshold regime, R^{ob} reduces to the classical reproduction number, and the dominant-eigenvector structure re-emerges.

For example, right after Eq. 3 we now write:

We emphasize that $\Rob$ is not a community-specific analogue of the spectral radius of R, nor an eigenvector-based sensitivity measure of $\Rref$. The spectral radius $\Rref=\rho(R)$ characterizes the linearized asymptotic dynamics of the epidemic and determines whether a system-level diffusion can be sustained. By contrast, $\Rob$, defined from the extinction probability of a branching process, is a nonlinear, seed-specific quantity: first the coupled fixed-point problem for the epidemic probabilities is solved, and then the resulting p_i is mapped onto the scalar reproduction ratio that an isolated community would need to have the same survival probability. A closed-form analytical solution of p_i, and thus $\Rob_i$ is possible only in the case of isolated communities by means of the Lambert-W function. In the spatially coupled systems the epidemic probabilities are determined by the full multidimensional fixed-point system. The previously discussed two-community system further illustrates the distinction between $\Rob_i$ and standard next-generation-matrix quantities: in that example $\Rref=R_{jj}$ is unchanged by the one-way coupling R_{ji}, whereas the outbreak probability p_i, and therefore $\Rob_i$, changes with R_{ji}.

Regarding the code accessibility, I congratulate the authors in making their code and data public and in a neat noteboke form. However, the code in its current format is unable to be run, with missing data files and no useful readme file information. The repo currently only exposes the notebook, one workbook, one CSV, and a one-line README, so it is not a complete runnable project by itself. A fresh Run All fails for two separate reasons:
(1) the main imports are in cell 3, but cell 0 tries to use pd.read_csv(...), so a clean notebook run hits the import-order problem first
(2) once that is fixed, the next failure is missing input data, starting with ./Italy_colocation.csv.

I suggest the authors upload the missing data files, ensure the code is runnable from a clean computer, with a top to bottom workflow and develop a useful readme file. Also Can I suggest that at least for publication that you ensure that the code is available on GitHub as a frozen tagged release representing the final analysis snapshot used for this manuscript.

We agree that ensuring full reproducibility is essential, and we have taken several steps to address these issues.

First, we have reorganized the notebook to ensure that it can be executed from top to bottom on a clean environment. In particular, all required imports are now placed at the beginning of the notebook, and the execution order has been corrected.

Second, we have improved the documentation by adding a detailed README file that describes the workflow, required dependencies, and instructions for running the analysis.

Regarding data availability, the Colocation Maps are available only upon individual request to and agreement with the Meta AI for Good program (https://ai.meta.com/ai-for-good/). We now explicitly provide this information in the repository and manuscript.

To ensure that the repository remains runnable and illustrative of the framework, we provide the full implementation of the R^{ob} methodology together with executable toy examples and simulation workflows.

Some spatial contact datasets used in the manuscript, such as the Meta Colocation Maps, are subject to external data-sharing agreements and therefore cannot be redistributed directly in the repository. However, the repository includes all core functions, simulation engines, and example workflows necessary to compute epidemic probabilities and R^{ob} from arbitrary reproduction operators.

Finally, we will maintain a public GitHub repository containing the implementation associated with this manuscript, including a tagged release corresponding to the published version of the framework.
