# Supplementary material for: Redefining and estimating the early-phase reproduction ratio for epidemic outbreaks in spatially structured populations
Source: PLoS Comput Biol. 2026 Jul 1;22(7):e1014425. doi: 10.1371/journal.pcbi.1014425 (PMC13349308; doi:10.1371/journal.pcbi.1014425)
Supplement: Attachment — Submitted filename: Review.docx [file pcbi.1014425.s002.docx]

In the study by Wang and Valdno, the authors define an outbreak reproduction number that takes into account spatial heterogeneity. They use colocation data and show that various locations have spatial heterogeneity I their transmission potential. Furthermore, they apply their reproduction number to early outbreak data to estimate locations at higher risk. The study is interesting, rigorous and well-written overall. I do have some questions and comments that should be answered before it is worthy of publication, in my opinion.

- The metrics used to investigate the usefulness of the proposed method. As you state, the main point of the paper is “…quantifying epidemic outbreak risk in spatially structured populations…”. However, there is no direct comparison between your and other methods, or even just a direct quantification of the precision of your method, for the probability of outbreak estimate. I think that it is worth performing simulations and using real data to see how well you can estimate outbreak probabilities under various conditions, rather than just estimating reproduction numbers.
- If you have several levels of spatial resolution (e.g. neighborhood, city, county), how would you choose the right one? Is there some criterion in your framework to quantify this bias-variance trade-off? This should probably be demonstrated, but at least discussed.
- You explain why a Poisson process is reasonable, despite common overdispersion, because overdispersion may be dealt with by your stratification of the population. However, this does not include cases of superspreaders, even in mostly homogenous populations (e.g. a fast-food worker sneezing on hundreds of burgers every day). Although you somewhat discuss this, it is a bit downplayed in my opinion, since there is a lot of evidence that superspreaders may play a major role in outbreak dynamics. I think that some simulations showing that your framework holds when introducing superspreaders would be beneficial.
- Are your estimates correct if the infectious period is not exponential? There are some works showing that the probability of an outbreak differs, and may be underestimated, if the infectious period is e.g. gamma distributed instead of exponential (which is quite common in real-world settings).
- What happens when the spatial contact data is noisy or biased, as may well be the case quite often? Perhaps worth pursuing in simulations, when the ground truth is known.
- Are there other estimates of R that summarize local information from heterogenous groups? prior work is worth referring to and comparing against.
- "We now show that Rob behaves consistently as a reproduction ratio by demonstrating that it reduces to known quantities in limiting cases, and has the same critical behavior. First, the epidemic threshold of the system (Rref = 1) correctly maps to Rob = 1." Does this have to be the case? Why limit yourself to this?
- Why do you have consistent overestimation of R (as in figure 3)?
- The order of the legend of figure 6b is not the same as figure 6a.
- Figures are not consistently referred to (e.g. Figure 5 and Fig. 5), and I can’t see figure 6 mentioned in the text.
